# Administration of ON 01210.Na after exposure to ionizing radiation protects bone marrow cells by attenuating DNA damage response

**DOI:** 10.1186/1748-717X-7-6

**Published:** 2012-01-20

**Authors:** Shubhankar Suman, Manoj Maniar, Albert J Fornace, Kamal Datta

**Affiliations:** 1 Department of Biochemistry and Molecular & Cell Biology, Georgetown University Medical Center, Research Building, Room E518, 3970 Reservoir Rd., NW, Washington, DC 20057-1468, USA; 2 Onconova Therapeutics Inc., Newton, PA 18940, USA

**Keywords:** Radiation toxicity, hematopoietic toxicity, ON 01210.Na, Ex-RAD, radiation mitigation, DNA damage.

## Abstract

**Background:**

Ionizing radiation-induced hematopoietic injury could occur either due to accidental exposure or due to diagnostic and therapeutic interventions. Currently there is no approved drug to mitigate radiation toxicity in hematopoietic cells. This study investigates the potential of ON 01210.Na, a chlorobenzylsulfone derivative, in ameliorating radiation-induced hematopoietic toxicity when administered after exposure to radiation. We also investigate the molecular mechanisms underlying this activity.

**Methods:**

Male C3H/HeN mice (n = 5 mice per group; 6-8 weeks old) were exposed to a sub-lethal dose (5 Gy) of γ radiation using a ^137^Cs source at a dose rate of 0.77 Gy/min. Two doses of ON 01210.Na (500 mg/kg body weight) were administered subcutaneously at 24 h and 36 h after radiation exposure. Mitigation of hematopoietic toxicity by ON 01210.Na was investigated by peripheral white blood cell (WBC) and platelet counts at 3, 7, 21, and 28 d after radiation exposure. Granulocyte macrophage colony forming unit (GM-CFU) assay was done using isolated bone marrow cells, and terminal deoxynucleotidyl transferase dUTP nick end-labeling (TUNEL) was performed on bone marrow sections at 7 d post-exposure. The DNA damage response pathway involving ataxia telangiectasia mutated (ATM) and p53 was investigated by Western blot in bone marrow cells at 7 d post-exposure.

**Results:**

Compared to the vehicle, ON 01210.Na treated mice showed accelerated recovery of peripheral WBC and platelet counts. Post-irradiation treatment of mice with ON 01210.Na also resulted in higher GM-CFU counts. The mitigation effects were accompanied by attenuation of ATM-p53-dependent DNA damage response in the bone marrow cells of ON 01210.Na treated mice. Both phospho-ATM and phospho-p53 were significantly lower in the bone marrow cells of ON 01210.Na treated than in vehicle treated mice. Furthermore, the Bcl2:Bax ratio was higher in the drug treated mice than the vehicle treated groups.

**Conclusions:**

ON 01210.Na treatment significantly mitigated the hematopoietic toxicity induced by a sub-lethal radiation dose. Mechanistically, attenuation of ATM-p53 mediated DNA damage response by ON 01210.Na is contributing to the mitigation of radiation-induced hematopoietic toxicity.

## Background

In addition to therapeutic and diagnostic interventions, exposure to sub-lethal doses of radiation to civilian population may occur during radiological accidents or terror attacks [1,2]. Depending on the duration of exposure, the area exposed, and the dose received, radiation exposure in the immediate aftermath could lead to a myriad of deleterious effects, including acute radiation syndrome (ARS) [3]. ARS is a well-defined dose-dependent pattern of organ damage, mainly affecting tissues with rapidly proliferating cells [4,5]. ARS includes hematopoietic syndrome (1 Gy to 8 Gy), gastrointestinal syndrome (> 8 Gy) and cardiovascular/CNS syndrome (> 20 Gy) and follows well defined pathologies [5,6]. Acute radiation exposure in the range between 1 and 8 Gy leads to a drop in circulating blood cells [7]. At the higher end of this radiation dose range, blood cell counts continue to decline due to the demise of bone marrow stem/progenitor cells leading to lethality. Blood cell counts also decline at lower doses, until surviving precursor cells proliferate to restore homeostasis [5]. During this period of declining bone marrow cells, individuals are at an increased risk of infection and hemorrhage [8]. A number of plant/herbal products, like *Hippophae rhamnoides and Mentha arvensis *and chemical entities, like amifostin and vitamin E related products, have been shown to modulate radiation toxicity. However, very few have been shown to have radiomitigator properties and fewer have been shown to improve hematological parameters [9,10]. Therefore, therapeutic agents mitigating radiation-induced decrease in bone marrow cells could play an important role not only in emergency situations but also in minimizing the radiation toxicity to bone marrow during radiation therapy or diagnostic procedures.

The tumor suppressor p53 plays a vital role in radiation-induced DNA damage response (DDR) and cell death [11]. The DDR starts with the sensing of the DNA damage, which is then conveyed to effectors via signal transducers. The ataxia telangiectasia mutated (ATM) protein is a well-established sensor of DNA damage and gets activated by autophosphorylation on sensing DNA damage [12]. Activated ATM in turn phosphorylates its downstream target p53, which is considered a signal transducer of DDR, to effectors like Bcl2-associated X protein (Bax), p21, and B-cell lymphoma 2 (Bcl2), to either induce cell cycle arrest for repair or initiate programmed cell death if the damage is beyond repair. It is important to note here that phosphorylation of p53 leads not only to its activation but also to its stabilization by minimizing its interaction with murine double minute 2 (MDM2), a negative regulator of p53 [13]. DNA damage-induced activation of p53 is known to regulate the transcription of two major cell death regulators: anti-apoptotic Bcl2 and pro-apoptotic Bax via down-regulation and trans-activation, respectively [11,14-16]. Bcl2 inhibits apoptosis by antagonizing Bax oligomerization, which is indispensible for Bax-mediated apoptosis [17]. In previous *in vitro *studies ON 01210.Na has been reported to reduce the level of p53 and its target p21 and Bax, when given prophylactically before radiation exposure [18]. Here we report that administration of 2 doses of ON 01210.Na at 24 and 36 h after radiation exposure augmented hematopoietic cell survival through reduction in DNA damage and damage response. Our results also indicate that increased clonogenic survival of bone marrow cells observed in ON 01210.Na treated mice was due to attenuation of p53-mediated apoptotic response.

## Methods

### Mice and radiation

Six to eight week old C3H/HeN male mice were purchased from Charles River Laboratories (Wilmington, MA, USA) and were housed in the Georgetown University's (GU) AAALACI (Association for Assessment and Accreditation of Laboratory and Animal Care International) accredited facility. All the animal procedures were performed according to protocols approved by the Georgetown University Animal Care and Use Committee (GUACUC), and terminal anesthesia with CO_2 _was used for collection of tissue and blood samples from these mice. For irradiation, mice were placed in a circular pie shaped, well-ventilated plastic mouse holder, and a ^137^Cs source (dose rate 0.77 Gy/min) was used as γ radiation source. Effects in irradiated+ON 01210.Na treated groups were compared to those in the radiation only and the radiation+vehicle treated groups and are shown in the results. The unirradiated control, drug only group, and vehicle only group acted as additional control groups. All irradiation groups were exposed to 5 Gy of γ radiation, control groups were sham irradiated and irradiation and experiments were repeated two times.

### ON 01210.Na formulation and administration

ON 01210.Na (Ex-RAD), a chlorobenzylsulfone derivative developed by Onconova Theraputics (Newtown, PA, USA) as a radioprotector and mitigator, was described earlier [18-20]. ON 01210.Na (500 mg/kg) was administered subcutaneously (SC), using 1 mL sterile syringe with a 25G needle at 24 and 36 h after 5 Gy radiation exposure.

### Peripheral white blood cell (WBC) and platelet counts

Blood samples (n = 5 mice per group per time point) were collected by cardiac puncture in ethylenediaminetetra acetic acid (EDTA) tubes after terminal CO_2 _anesthesia at 3, 7, 21, and 28 d after radiation exposure and subjected to complete blood count. White blood cell (WBC) counts, neutrophil counts, and monocyte counts are presented as absolute count, and platelet counts are presented as percent of normal count (1.2 × 10^6 ^per μL of blood, ± 16.1 standard error of mean).

### Bone marrow histopathology

Bone marrow (n = 5 mice per group) was used for histopathologic analysis. Femurs were surgically removed from each mouse at 7 d after 5 Gy radiation exposure, fixed in 10% buffered formalin for 48 h, decalcified, paraffin embedded, and 5 μm thick sections were stained with hematoxylin and eosin (H&E) using standard procedures. Unstained sections were used for terminal deoxynucleotidyl transferase dUTP nick end-labeling (TUNEL) assay. Bone marrow cellularity in H&E stained sections was semiquantitatively scored (in 5 mice from each group) by counting nucleated cells in a 16-square (1 cm^2 ^each) grid in randomly chosen 5 high-power (40×) microscopic fields for each section (3 sections from each mouse) as described earlier [21]. While plotting the results, control section cellularity was considered 100 percent. Megakaryocytes were also evaluated by a semi-quantitative analysis of three adjacent high-power (40×) microscopic fields (n = 5 and 3 sections from each mouse were scored).

### Granulocyte Macrophage-Colony Forming Unit (GM-CFU) assay

Mice (n = 5 mice per group) were euthanized at 7 d after radiation exposure. Under aseptic conditions femurs were excised, ends opened, and bone marrow cells collected by flushing. For flushing, Iscove's modified Dulbecco's medium (IMDM) (StemCell Technologies, Vancouver, BC, Canada) supplemented with 5% fetal bovine serum (FBS) was used with sterile syringes and 25G needles. Flushed bone marrow from each femur was pipetted up and down to prepare a single cell suspension and passed through 70 micron nylon meshes (BD Biosciences, Sparks, MD, USA). Isolated cells were counted using a cell counter (Beckman Coulter, Brea, CA, USA), and from each femur 2.5 × 10^4 ^cells/mL were plated in triplicate in ultra-low attachment 60 mm dishes (Corning, NY, USA) using methocult (M3534, StemCell Technologies) medium supplemented with 10 ng/mL granulocyte macrophage colony stimulating factor (GM-CSF) (StemCell Technologies). The plates were incubated at 37°C in 5% CO_2 _and ≥ 95% humidity for 7 d, and colonies were counted using a dissecting microscope (Leica, Wetzlar, Germany).

### Apoptosis detection in bone marrow and spleen

DNA damage and cell death in bone marrow and spleen cells (n = 5 mice per group) were detected, using the ApopTag Plus Peroxidase *in situ *apoptosis detection kit (S7101, Millipore, Billerica, MA, USA) according to manufacturer's instruction. Briefly, tissue sections were deparaffinized and pretreated with Proteinase-K solution (20 μg/mL) at room temperature for 15 min. The endogenous peroxidase activity was quenched using 3% hydrogen peroxide in phosphate buffered saline (PBS) at room temperature. Following incubation with terminal deoxynucleotidal transferase (TdT) at 37°C for 1 h, the apoptotic cells were visualized under a bright field microscope by a diaminobenzidine (DAB) based detection system supplied with the kit, and sections were counterstained using methyl green (Trevigen, Gaithersburg, MD, USA) nuclear stain. TUNEL positive cells were counted in 5 randomly chosen high power fields (40×), and counts from 3 sections from each mouse were used for statistical analysis.

### Western blot

Bone marrow cells were isolated, as per the protocol described in the previous section, and cells from 5 mice were pooled for the Western blot analysis. Cells were lysed in ice-cold protein extraction buffer (0.5% Sodium deoxycholate, 0.5% NP-40, 10 mM EDTA in PBS) containing protease inhibitor cocktail (Sigma, St. Louis, MO, USA). The homogenate was centrifuged at 12000 xg at 4°C for 10 min and supernatant was collected. The Bradford protein assay was used to quantify the protein concentration in respective samples. Equal amounts of protein samples were mixed with the appropriate volume of Laemmli's sample buffer (6× solution: 375 mM Tris-HCl (pH = 6.8), 6% sodium dodecyl sulphate (SDS), 48% Glycerol, 9% β-Mercapto-ethanol and 0.03% bromophenol blue), heated at 95°C for 5 min, and were resolved on SDS- polyacrylamide gel electrophoresis (PAGE). Proteins were transferred onto a polyvinylidine fluoride (PVDF) membrane, blocked with 5% milk in tris-buffered saline with 0.1% Tween (TBST), and incubated with appropriate primary antibody (p-ATM (1:100, Sc-47739, Clone-10H11-E12); p53 (1:500, Sc-98, Clone-1801); p-p53 (1:200, Sc-18078, Clone-mSer20); Bcl-2 (1:250, Sc-7382, Clone-C-2); Bax (1:250, Sc-7480, Clone-B-9) and β-actin (1:2000, Sc-47778, Clone-C4) from Santa Cruz Biotechnology, Santa Cruz, CA, USA. Western blot membranes were developed with horseradish peroxidase (HRP) conjugated secondary antibody and enhanced chemiluminescence (ECL) detection system (Cat# 34080, Thermo Fisher Scientific, Rockford, IL, USA). Images were captured on photographic films and scanned. Results from a representative experiment are displayed. Scanned images of the Western blots were quantified by ImageJ v4.44 software using the previously described protocol [22]. Briefly, scanned images were opened in ImageJ, and bands were selected using the rectangular selection tool to generate band profile plots. Normalized band intensity was generated using β-actin band intensity in respective columns.

### Statistical analysis

Statistical analysis to find significance between two groups was performed using two tailed paired Student's t-test, and p < 0.05 was taken as statistically significant. Error bars represent ± standard error of mean (SEM).

## Results

### Accelerated recovery of peripheral blood cell count and increased clonogenic survival of bone marrow progenitors in ON 01210.Na treated group

**Figure 1:**
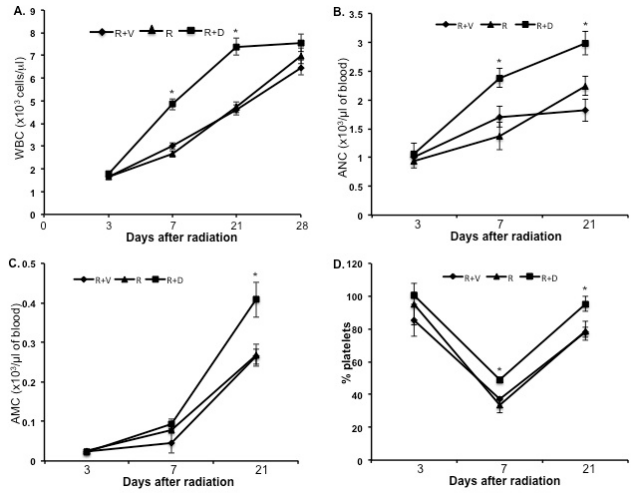
**Post-exposure peripheral blood cell count with or without ON 01210.Na treatment**. A) Absolute peripheral WBC counts. B) Absolute neutrophil count (ANC). C) Absolute monocyte counts (AMC). D) Platelet count expressed as percent of normal count. R+V: 5 Gy radiation+vehicle; R: 5 Gy radiation; R+D: 5 Gy radiation+drug (ON 01210.Na). *p < 0.05 compared to radiation+vehicle.

**Figure 2:**
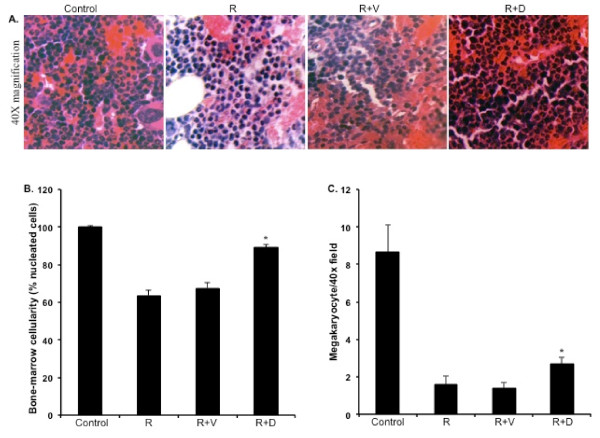
**Quantification of bone marrow cellularity in H&E stained sections at 7 d post-radiation**. A) Photomicrograph showing H&E stained sections of bone marrow at 40× magnification. B) Relative quantification of total bone marrow cellularity. *p < 0.004 compared to radiation+vehicle (R+V). C) Megakaryocyte counts in H&E stained bone marrow sections. *p < 0.02 compared to radiation+vehicle. Control: no radiation, ON 01210.Na or vehicle; R: 5 Gy radiation; R+V: 5 Gy radiation+vehicle; R+D: 5 Gy radiation+drug (ON 01210.Na).

**Figure 3:**
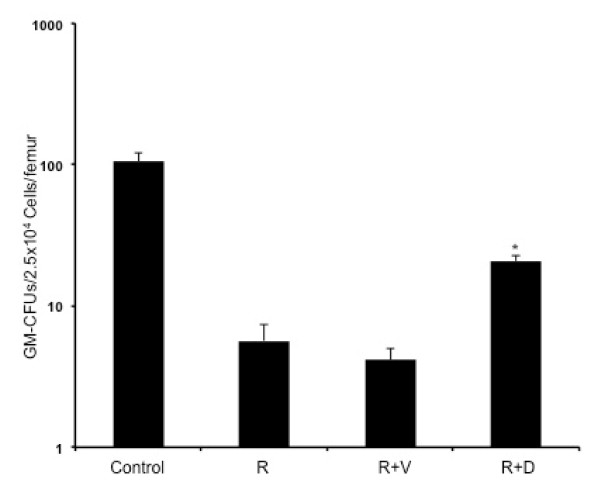
**Granulocyte-macrophage colony forming units (GM-CFU) assay of bone marrow cells at 7 d post-radiation**. *p < 0.005 compared to radiation+vehicle (R+V). Control: no radiation, ON 01210.Na or vehicle; R: 5 Gy radiation; R+V: 5 Gy radiation+vehicle; R+D: 5 Gy radiation+drug (ON 01210.Na).

The WBC count at 3 d showed uniform reduction in all the irradiated groups. However, at 7 and 21 d significantly higher counts were observed in mice treated with ON 01210.Na (compared to respective radiation+vehicle groups p < 0.04 for 7 d and p < 0.01 for 21 d) (Figure 1A). A significant difference in absolute neutrophil count (ANC) was also observed between the ON 01210.Na treated and the vehicle treated groups at 7 and 21 d (p < 0.05 for both of the time points) (Figure 1B). When absolute monocyte counts (AMC) were compared between the ON 01210.Na and the vehicle treated groups, a significant difference was observed at 21 d (p < 0.05) (Figure 1C). In contrast to WBC count, platelet count was decreased at 7 d and recovery was observed at 21 d. However, at 7 d the count was significantly higher in the ON 01210.Na treated group than the vehicle treated group (p < 0.05), and at 21 d the ON 01210.Na treated group showed greater recovery than the vehicle group (p < 0.05) (Figure 1D). Furthermore, total cellularity, as well as megakaryocyte counts in bone marrow sections, was significantly higher in the ON 01210.Na than in the vehicle treated mice (Figure 2A, B, and 2C). When given 24 h and 36 h after radiation exposure, the ON 01210.Na treated group also showed significantly enhanced clonogenic survival of bone marrow cells (p < 0.0005 compared to the radiation+vehicle group) (Figure 3).

### ON 01210.Na treatment showed reduction in apoptotic cells in bone marrow and spleen

**Figure 4:**
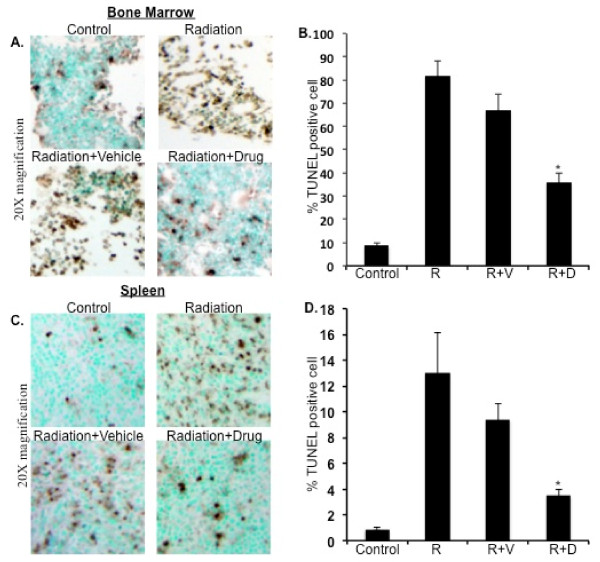
**TUNEL staining of bone marrow and spleen sections at 7 d post-radiation**. A) Photomicrograph showing TUNEL staining of bone marrow sections at 20× magnification. B) Quantification of TUNEL positive cells in bone marrow sections. *p < 0.01 compared to radiation+vehicle (R+V). C) Photomicrograph showing TUNEL staining of spleen sections at 20× magnification. D) Quantification of TUNEL positive cells in spleen sections. *p < 0.05 compared to radiation+vehicle. Control: no radiation, ON 01210.Na or vehicle; R: 5 Gy radiation; R+V: 5 Gy radiation+vehicle; R+D: 5 Gy radiation+drug (ON 01210.Na).

TUNEL assay on bone marrow and spleen sections from the ON 01210.Na treated groups showed fewer apoptotic cells than the vehicle treated group (Figure 4A and 4C). Quantification of TUNEL positive cells indicating apoptosis showed significantly lower counts in the ON 01210.Na treated group than the vehicle treated group in both the bone marrow and spleen samples (Figure 4B and 4D; p < 0.01 for bone marrow and p < 0.05 for spleen compared to radiation+vehicle groups).

### Attenuated DNA damage response in bone marrow cells of ON 01210.Na treated mice

**Figure 5:**
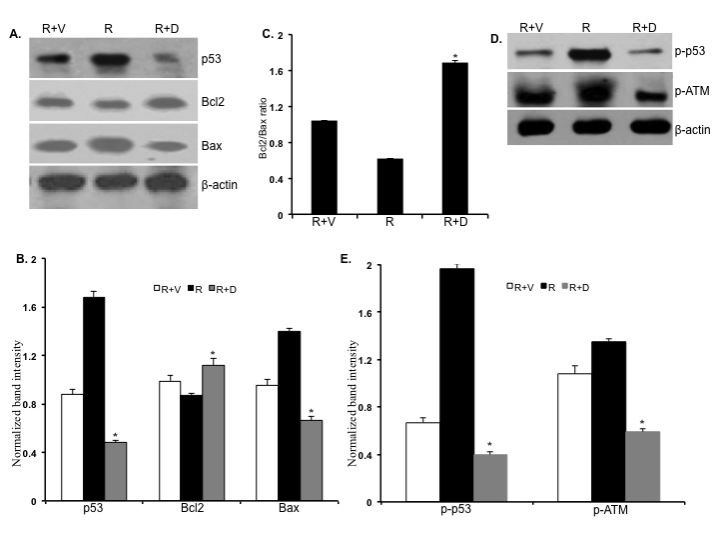
**Western blot analysis of DNA damage response in bone marrow cells at 7 d post-radiation**. A) Scanned images showing Western blot of p53, Bcl2, and Bax. B) Densitometric quantification of p53, Bcl2, and Bax. *p < 0.002 for p53, < 0.001 for Bax, and < 0.007 for Bcl2 compared to respective radiation+vehicle groups C) Bcl2/Bax ratio. *p < 0.005 compared to radiation+vehicle. D) Western blot images of phospho-ATM and phospho-p53. E) Densitometric quantification of phospho-ATM and phospho-p53. *p < 0.0007 for p-ATM and < 0.0002 for p-p53 compared to radiation+vehicle treated mice. R+V: 5 Gy radiation+vehicle; R: 5 Gy radiation; R+D: 5 Gy radiation+drug (ON 01210.Na).

The DNA damage response pathway involving ATM and p53 was assessed by Western blot analysis. Compared to vehicle treated groups, we observed a significant reduction of total p53, phospho-ATM, and phospho-p53 levels in ON 01210.Na treated bone marrow cells at 7 d post-radiation (Figure 5A and 5D). Decrease of the p53 level in ON 01210.Na treated mice was associated with increase in anti-apoptotic Bcl2 and decrease in pro-apoptotic Bax (Figure 5A). Quantification of Western blots showed significant decrease in total p53 and Bax but increase in Bcl2 (Figure 5B; p < 0.002 for p53, < 0.001 for Bax, and < 0.007 for Bcl2 compared to radiation+vehicle groups). Interestingly, the Bcl2:Bax ratio was markedly greater in the ON 01210.Na treated than the vehicle treated mice (Figure 5C). Furthermore, quantification of phospho-ATM and phospho-p53 showed significant decrease in the ON 01210.Na treated mice (Figure 5E; p < 0.0007 for p-ATM and < 0.0002 for the p-p53 compared to the radiation+vehicle treated mice).

## Discussion

Terrorist threats and recent accidents in nuclear installations emphasize the need to develop strategies to mitigate radiation toxicity [23]. Mitigation of radiation toxicity also has implications for radiation therapy [3]. In this initial investigation we have shown that two doses of ON 01210.Na, administered 24 h and 36 h after radiation exposure, could significantly mitigate radiation-induced hematopoietic toxicity. Mechanistically, ON 01210.Na treatment resulted in diminished radiation-induced DDR and apoptosis. Overall, ON 01210.Na was able to accelerate the post-exposure hematopoietic recovery process as evidenced by a greater increase in peripheral WBC and platelet counts, higher bone marrow cellularity, and higher number of GM-CFUs in the drug treated groups than in the vehicle groups.

Most of the post-radiation hematopoietic morbidity and mortality is attributed to infection and hemorrhage due to leukopenia and thrombocytopenia resulting from loss of bone marrow cells. Our time course study ranging from 3 to 28 d demonstrated faster recovery of radiation-induced WBC cell loss in the ON 01210.Na than in the vehicle treated groups. Higher numbers of peripheral WBC counts in the ON 01210.Na treated animals led us to believe that the drug is aiding in quicker regeneration and turnover of bone marrow cells than the vehicle groups through proliferation of surviving precursor cells. Importantly, at 7 d and at 21 d in the ON 01210.Na groups, the absolute neutrophil counts were significantly higher which would equip these animals to resist any potential post-radiation infection better than the vehicle treated groups. Furthermore, the ON 01210.Na treated groups at both these time points showed greater percent of platelets than the vehicle groups, which would minimize not only post-radiation infection but would also reduce the risk of hemorrhagic events.

Bone marrow progenitor cells with an average lower radiosensitivity than the more primitive hematopoietic precursor cells are a rapidly cycling cell population and are capable of forming colonies in culture (reviewed by [24]). Our results from the GM-CFU assay support the notion that ON 01210.Na is enhancing the recovery and regeneration of the progenitor cells, which survived initial radiation-induced damage. Mechanistic investigation further supports our hypothesis that ON 01210.Na is accelerating recovery/regeneration of bone marrow cells due to attenuation of DDR. The ON 01210.Na, through lowering of phospho-ATM and p53, along with a higher Bcl2/Bax ratio, could be aiding in the proliferation of the surviving cells. Our results showing diminution of p53 level, along with increase in Bcl2 and decrease in Bax level, demonstrate the mechanism of ON 01210.Na mediated radiation mitigation and is important for the management of radiation exposure to healthy tissues.

Bone marrow and spleen are important in maintaining peripheral blood cell pool and proper functioning of the immune system. Thus, radiation damage to these vital organs can affect hematopoiesis as well as immune defense, which are critical determinant of post-exposure morbidity and mortality [25,26]. Although chemical entities and herbal preparations have been investigated for their radioprotection and radiomitigation properties, we are yet to have an approved pharmacological agent to counter radiation damage to critical tissues, like bone marrow, and to reduce subsequent morbidity and mortality [27]. Compounds like statins and palifermin has been shown to mitigate radiation mucositis and enteropathy, and herbal preparations from *Hippophae rhamnoides *and *Mentha arvensis *have shown significant hematopoietic protection [3,9,10]. However, there are presently no approved agents available to mitigate radiation-induced hematopoietic toxicity. In a post-exposure scenario, administration of ON 01210.Na was able to reduce the number of apoptotic cells (TUNEL positive) in both bone marrow and spleen, indicating lower cell death than that with vehicle treatment. Our Western blot results suggest that lessening of cell death in bone marrow and spleen has to be p53 mediated and is dependent on the activated ATM. Although we did not observe any alterations in total ATM levels (data not shown), we did find significantly lower levels of activated phospho-ATM and, consequently, significantly reduced phospho-p53 in the ON 01210.Na treated bone marrow cells. Stability of p53 is dependent on its phosphorylation status [12]. We believe that decreased total p53 in bone marrow cells is due to reduced phosphorylation of p53, allowing enhanced interaction with MDM2, leading to increased ubiquitinylation and subsequent degradation. Pro-apoptotic Bax and anti-apoptotic Bcl2 are under the regulation of tumor suppressor p53. Antagonizing Bax and enhancing Bcl2 has been shown to confer resistance on cells to ionizing radiation [28,29]. Reduction in activated ATM and p53 levels leading to a decrease in DDR signal, is playing a role in enhanced hematopoietic recovery in ON 01210.Na treated mice.

## Conclusions

Taken together, we conclude that the post-exposure administration of ON 01210.Na enhances the recovery of hematopoietic cells by employing a mechanism that not only attenuates DNA damage sensing and damage signal transduction but also alters levels of effectors like Bax and Bcl2.

## Competing interests notification

Dr. Manoj Maniar is employed at Onconova Therapeutics, Inc. No other authors have financial obligations to Onconova Therapeutics, Inc.

## Authors' contributions

SS: executed experiments and analyzed and organized results; KD: planned and executed experiments, analyzed results, and prepared the manuscript; AJF: participated in preparing the manuscript; MM: planned experiments and participated in preparing the manuscript. All authors read and approved this manuscript.
